# Association of triglycerides to high-density lipoprotein cholesterol ratio to identify future prediabetes and type 2 diabetes mellitus: over one-decade follow-up in the Iranian population

**DOI:** 10.1186/s13098-023-00988-0

**Published:** 2023-02-02

**Authors:** Maryam Tohidi, Samaneh Asgari, Abdolreza Chary, Siavash Safiee, Fereidoun Azizi, Farzad Hadaegh

**Affiliations:** 1 Prevention of Metabolic Disorders Research Center, Research Institute for Endocrine Sciences, Shahid Beheshti University of Medical Sciences, P.O.Box 19395-4763, Tehran, Islamic Republic of Iran; 2 Endocrine Research Center, Research Institute for Endocrine Sciences, Shahid Beheshti University of Medical Sciences, Tehran, Iran

**Keywords:** Dysglycemia, Prediabetes, Type 2 diabetes mellitus, Triglycerides, High-density lipoprotein cholesterol, Ratio

## Abstract

**Background:**

To determine the association between triglyceride to high-density lipoprotein cholesterol ratio (TG/HDL-C) for identifying subjects at risk of incident prediabetes and type 2 diabetes mellitus (T2DM).

**Methods:**

In 5064 subjects (men = 2247) aged ≥ 20 years, using Cox proportional hazards regression analyses, the associations of TG/HDL-C with incident prediabetes and T2DM were examined among normoglycemic men and women. Furthermore, the association of this lipid ratio with incident T2DM was also assessed among prediabetic subjects (n = 1414). The multivariable analyses were adjusted for age, body mass index, waist-to-height ratio, wrist circumference, systolic blood pressure, family history of T2DM, education level, history of cardiovascular diseases, and fasting plasma glucose (FPG).

**Results:**

During a median follow-up of 11.2 years, 2140 new cases of prediabetes (men = 1070) and 360 incident T2DM (men = 152) were identified among normoglycemic individuals. In the prediabetic population, 574 new cases of T2DM (men = 252) were developed. Among the whole population, compared to the first quartile (reference), higher quartiles of TG/HDL-C were significantly associated with higher risks of incident prediabetes and T2DM among normoglycemic individuals and incident T2DM in the prediabetic population (all *P* for trend < 0.001). The corresponding hazard ratios (HRs) and 95% confidence intervals (CIs) for the fourth quartiles were 1.37(1.20–1.58), 1.92(1.34–2.75), and 1.57(1.22–2.01), respectively. The sex-stratified analyses demonstrated similar significant associations in both sexes; however, TG/HDL-C lost its association with incident T2DM among prediabetic men. Among the normoglycemic population, 1 unit increase in TG/HDL-C was significantly associated with incident prediabetes and T2DM [1.02(1.00–1.03) and 1.06(1.03–1.08), respectively]. The corresponding value for incident T2DM in prediabetic individuals was 1.01(1.00–1.03). In a subgroup population having insulin data (n = 2897), the associations between TG/HDL-C and incident prediabetes and T2DM among normoglycemic individuals generally persisted even after replacing FPG with an index of insulin resistance (IR), i.e., homeostasis model assessment of IR (HOMA-IR) in the adjusted model.

**Conclusions:**

In conclusion, in the normoglycemic population, the increasing value of TG/HDL-C was unfavorably associated with incident prediabetes and T2DM, especially among women. Similarly, TG/HDL-C was associated with incident T2DM in prediabetic individuals. Generally, we found that the correlation between TG/HDL-C and different states of dysglycemia is independent of HOMA-IR.

**Supplementary Information:**

The online version contains supplementary material available at 10.1186/s13098-023-00988-0.

## Introduction

Dysglycemia has a well-known natural history with a continuum starting from the asymptomatic phase, including prediabetes and preclinical latent diabetes, to clinically diagnosed diabetes mellitus (DM) [1]. As an important dysglycemic state, type 2 DM (T2DM) has an increasing prevalence worldwide, with a global prevalence of 8.8% in 2017, and it is estimated that 642 million subjects will develop T2DM by 2040 [2]. The annual incidence of T2DM is reported to be over 1% in the Iranian population [3]. Prediabetes, another dysglycemic state, is currently a global health problem. The reported prevalence of prediabetes varies due to different criteria that were used to define this intermediate hyperglycemic state, including impaired fasting glucose (IFG), impaired glucose tolerance (IGT), or borderline elevation of glycated hemoglobin (HbA1C) [4]. The global prevalence of prediabetes, defined only as IGT, was estimated to be 7.5% in 2019 and expected to reach 8.0% and 8.6% by 2030 and 2045, respectively [5]. According to an Iranian national population-based study, the prevalence of IFG was reported to be 16.8% in 2008 [6]. Moreover, every year more than 4% of the Iranian population develop prediabetes [7]. Prediabetes and T2DM are associated with a high burden of non-communicable diseases, including cardiovascular diseases (CVD), chronic kidney disease, and all-cause mortality [8, 9].

Insulin resistance (IR) is the leading underlying cause in the pathogenesis of prediabetes and T2DM [10]. The reference method for assessing IR, i.e., hyperinsulinemic-euglycemic clamp, as a complicated, expensive, and burdensome technique, is not feasible for epidemiologic studies [11]. Although homeostasis model assessment of IR (HOMA-IR) is suggested as a proper index of IR, it needs laboratory measurement of insulin level that is not available in all healthcare services or is expensive for routine practice [12]. Therefore, simple markers associated with IR may help identify individuals at high risk for prediabetes and T2DM.

Dyslipidemia, mainly as elevated triglycerides (TG) and low level of high-density lipoprotein cholesterol (HDL-C), is among the basic characteristics of conditions associated with IR, and it was shown that TG/HDL-C ratio could be used as a surrogate of IR [13–15]. Limited data are available regarding the association between TG/HDL-C and incident prediabetes and T2DM in the frame of prospective cohort studies. Most studies on the association between TG/HDL-C and IR, metabolic syndrome, prediabetes, and diabetes were performed with retrospective cohort or cross-sectional designs [13, 16–20]. Previously, in the Tehran Lipid and Glucose Study (TLGS), we showed that TG/HDL-C is an independent risk factor for T2DM during about 6-year follow-up among the nondiabetic population [21]. Moreover, we also found that TG/HDL-C is an important predictor of prediabetes only among Tehranian women [7]. According to previous studies, TG/HDL-C ratio was suggested as a diagnostic or screening tool for different conditions, including those at risk of metabolic syndrome, IR, T2DM, and identifying those at risk of chronic kidney disease or CVD [10, 22–27].

We aimed to extend our previous studies in three ways in the current study. First, the associations between TG/HDL-C with incident prediabetes and T2DM among participants with normoglycemia [i.e., normal fasting glucose (NFG) and normal glucose tolerance (NGT)] were investigated. Second, the relationship between TG/HDL-C and incident T2DM was examined among individuals with prediabetes at baseline. Third, the association between TG/HDL-C and different states of dysglycemia in the presence of HOMA-IR was also assessed.

## Methods

### Study population

The current study is designed in the framework of the TLGS, a population-based prospective cohort conducted on a Tehranian urban population aged ≥ 3 years to determine the prevalence, incidence, and risk factors of non-communicable diseases.

The TLGS enrollment was carried out in two phases, the first phase [1999–2002, n = 15,005], the second phase [2002–2005, n = 3550] and is planned to keep on for at least 20 years with triennial follow-ups; the participants had consecutive follow-up visits (2002–2005, for those enrolled in the first phase), (2005–2008), (2008–2011), (2011–2015), and 2015–2018). The design and methodology of the TLGS have been reported elsewhere [28].

**Fig. 1:**
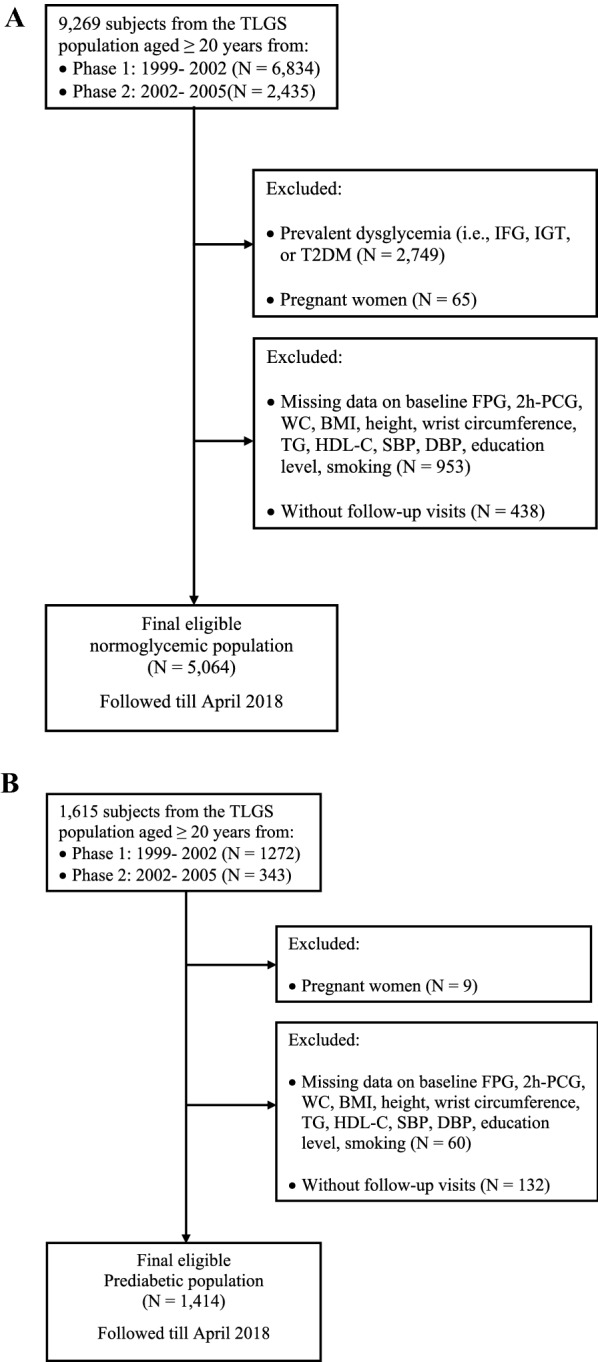
Flow diagram of the study participants. **A** Normoglycemic population to incident prediabetes or T2DM. **B** Prediabetic population to incident T2DM. *TLGS* Tehran lipids and glucose study, IFG impaired fasting glucose, IGT impaired glucose tolerance, *T2DM* type 2 diabetes mellitus, *N* number, *FPG* fasting plasma glucose, *2 h-PCG* 2-h post-challenge plasma glucose, *WC* waist circumference, *BMI* body mass index, *TG* triglycerides, *HDL-C* high-density lipoprotein cholesterol, *SBP* systolic blood pressure, *DBP* diastolic blood pressure

Figure 1 shows the detailed selection process of the study population for each outcome separately. For analysis of incident prediabetes and T2DM among participants with normoglycemia at baseline, of 9269 individuals aged ≥ 20 years (6834 from phase 1 and 2435 from phase 2), we excluded those with prediabetes or T2DM (i.e., IFG, IGT, or T2DM) at baseline (n = 2749), pregnant women (n = 65), and those without complete information on fasting plasma glucose (FPG), 2-h post-challenge plasma glucose (2 h-PCG), or other confounders (n = 953) considering overlapped features between numbers. After excluding those with missing data at the follow-up visits (n = 438), 5064 individuals (men = 2247) were eligible for the current study who followed till April 2018. For incident T2DM among the prediabetic population at baseline, after including 1615 prediabetic individuals aged ≥ 20 years (1272 from phase 1 and 343 from phase 2), we excluded pregnant women (n = 9), those without complete information on covariates (n = 60), or those without any follow-up (n = 132), 1414 prediabetic subjects (men = 647) were eligible for the current study. Moreover, as a sensitivity analysis, we examined the association between TG/HDL-C and incident prediabetes and T2DM in the presence of HOMA-IR in a subpopulation with insulin data (n = 2897).

The Institutional Review Board of the Research Institute for Endocrine Sciences (RIES), Shahid Beheshti University of Medical Sciences, Tehran, Iran, approved this study and all participants provided written informed consent.

### Clinical and laboratory measurement

In the baseline enrollment and triennial follow-up visits, the same parameters data through questionnaires and clinical and laboratory measurements were collected for all participants.

Trained interviewers using a pretested questionnaire, including questions about demographic, family history of DM (FH-DM), history of CVD, medication history, smoking habits, and education levels collected information.

To measure anthropometric parameters, subjects with light clothing and without shoes were examined. Weight was measured using a digital scale (Seca 707, Seca Corp; range 0·1–150 kg, sensitivity 0·1 kg). Height was measured with a tape meter in a standing position and shoulders in normal alignment. Body mass index (BMI) was calculated as weight (kg) divided into the square of height (${\mathrm{m}}^{2}$). Waist circumference (WC) at the umbilical was measured by a non-stretchable tape meter. To measure the wrist circumference at distal to the prominences of ulnar and radial bone, a tape meter was used while the subjects held the anterior surface of their right wrist up. After fifteen minutes of rest, systolic and diastolic blood pressures (SBP and DBP, respectively) were measured twice on the right arm by a standardized mercury sphygmomanometer (calibrated by the Iranian Institute of Standards and Industrial Researches), and blood pressure was considered a mean of these measurements.

For all subjects, after 12–14 h of overnight fasting, a venous blood sample was collected, and a standard oral glucose tolerance test was performed. FPG and 2 h-PCG were measured using an enzymatic colorimetric method with glucose oxidase. Total cholesterol (TC) was assayed using the enzymatic colorimetric method with cholesterol esterase and cholesterol oxidase. HDL-C was measured after precipitation of the apolipoprotein-B-containing lipoproteins with phosphotungstic acid. TG was assayed using an enzymatic colorimetric method with glycerol phosphate oxidase. These analyses were performed in the TLGS research laboratory on the same day as blood sampling using commercial kits (Pars Azmoon Inc., Tehran, Iran) and a Selectra 2 auto-analyzer (Vital Scientific, Spankeren, The Netherlands). TG/HDL-C was calculated by dividing TG by HDL-C. Assayed serum controls in two different concentrations (TruLab N and TruLab P; Pars Azmoon Inc.) were used to monitor the quality of measurements. The intra- and inter-assay coefficients of variation (CVs) both were 2.2% for glucose. For both TC and HDL-C, intra- and inter-assay CVs were 0.5 and 2%, respectively. Intra- and inter-assay CVs were 0.6 and 1.6% for TG, respectively.

### Definitions

The desired outcomes are prediabetes [(IFG (5.6 ≤ FPG < 7.0 mmol/L) or IGT (7.8 ≤ 2 h-PCG < 11.1 mmol/L], and T2DM (FPG ≥ 7.0 mmol/L or 2 h-PCG ≥ 11.1 mmol/L or using antidiabetic medications). Normoglycemia state includes NFG and NGT; NFG and NGT were defined as FPG < 5.6 mmol/L and 2 h-PCG < 7.8 mmol/L without antidiabetic medications, respectively.

HOMA-IR was calculated as HOMA-IR = fasting insulin (IU/mL) × fasting glucose (mmol/L)]/22.5 [29].

Levels of education were categorized into three groups based on self-reports: illiterate/primary school (≤ 6 years, as reference), 6–12 years, and ≥ 12 years. A current smoker (past/never smoker, as reference) was one who smokes cigarettes/pipes daily or occasionally. FH-DM was considered positive if the subject had at least one parent or sibling with diabetes.

### Statistics

Baseline characteristics of the study population were expressed as mean ± standard deviation (SD) and number (%) for categorical variables. The median [interquartile range (IQR)] was reported for covariates with a skewed distribution. A comparison of baseline characteristics between TG/HDL-C quartiles was made by the ANOVA test for normally distributed continuous variables, Kruskal–Wallis rank test for skewed variables, and the chi-squared test for categorical variables. Moreover, the comparisons between respondent versus non-respondent (i.e., those with missing data on baseline or lost to follow-up) were made by the Student’s t-test for normally distributed continuous variables, the Maan-Whitney u test for skewed variables, and the chi-squared test for categorical variables.

Cox proportional hazards models were used to evaluate the associations between one unit increment in TG/HDL-C and the incidence of either prediabetes or T2DM. We also determined the association between quartiles of TG/HDL-C with defined outcomes to find the possible linear associations. The event date for either prediabetes or T2DM was defined as the mid-time between the date of the follow-up visit at which the outcome was detected for the first time and the most recent follow-up visit before the diagnosis. For the censored and lost follow-up participants, the survival time was the interval between the first and the last observation dates. All analysis was done separately for men, women, and the whole population. A sequential modeling method was used to estimate the hazard ratios (HRs) with 95% confidence intervals (CIs) for TG/HDL-C both as continuous (per 1 unit increase) and quartile measures (first quartile as reference) in the development of each outcome in three models as; model 1: unadjusted model; model 2: adjusted for sex (in the whole population), age, BMI, waist-to-height ratio, wrist circumference, SBP, FH-DM, education levels, history of CVD, and model 3 further adjusted with baseline FPG. The confounders were selected according to the international guidelines and our previous studies [3, 4, 30]. Although TG/HDL-C is considered a surrogate of IR, as a sensitivity analysis, we also examined if the association between TG/HDL-C and incident prediabetes and T2DM persisted even after adjustment for HOMA-IR instead of FPG.

The proportional hazard assumption of the multivariable Cox models was assessed using Schoenfeld’s global test of residuals. All analyses were conducted using STATA version 14 SE (StataCorp, TX, USA), and a two-tailed p < 0.05 was considered significant.

## Results

The baseline characteristics of the study participants stratified by quartiles of TG/HDL-C in men and women have been presented in Tables 1 and 2, respectively. In both men and women, compared to the participants in the first quartile, those in the fourth quartile tended to be older and less educated (only women) but with higher BMI, WC, waist/height ratio, wrist circumference, FPG, 2 h-PCG, TG, TC, TG/HDL-C ratio, and blood pressure measures. Compared to non-respondent individuals, respondents were older and more obese with higher WC, waist-to-height ratio, wrist circumference, FPG, TC, TG, and TG/HDL-C, and lower levels of HDL-C. Furthermore, they had a lower prevalence of being a current smoker than non-respondent subjects (Additional file 1: Table S1).

**Table 1:** Baseline characteristics of the study population according to the TG/HDL-C categories among normoglycemic^†^ men

Variables	First (N = 565)	Second (N = 559)	Third (N = 564)	Fourth (N = 559)	*P* Value
	TG/HDL-C < 1.09	1.09 ≤ TG/HDL-C < 1.69	1.69 ≤ TG/HDL-C < 2.65	TG/HDL-C ≥ 2.65	
Age (years)	39.96 ± 16.3	42.04 ± 15.7	42.90 ± 13.9	42.26 ± 12.7	0.005
BMI (kg/m^2^)	23.76 ± 4.0	25.37 ± 3.8	26.52 ± 3.9	27.45 ± 3.5	< 0.001
WC (cm)	85.98 ± 10.7	90.50 ± 10.3	93.7 ± 9.8	95.93 ± 9.1	< 0.001
Height (cm)	171.48 ± 6.7	171.29 ± 6.9	170.69 ± 7.1	171.20 ± 6.8	0.001
Waist/Height	0.50 ± 0.07	0.53 ± 0.06	0.55 ± 0.06	0.56 ± 0.05	< 0.001
Wrist circumference (cm)	17.48 ± 1.10	17.78 ± 1.00	17.96 ± 1.00	18.12 ± 1.00	< 0.001
SBP (mmHg)	114.21 ± 15.6	115.73 ± 16.2	117.39 ± 14.9	117.59 ± 14.5	< 0.001
DBP (mmHg)	72.01 ± 10.3	73.44 ± 10.9	76.08 ± 10.0	76.60 ± 9.7	< 0.001
FPG (mmol/L)	4.82 ± 0.35	4.86 ± 0.36	4.89 ± 0.35	4.94 ± 0.34	< 0.001
2 h-PCG (mmol/L)	5.05 ± 1.16	5.17 ± 1.10	5.38 ± 1.20	5.50 ± 1.19	< 0.001
TC (mmol/L)	4.33 ± 0.94	4.74 ± 0.94	4.92 ± 0.97	5.17 ± 0.91	< 0.001
TG (mmol/L)	0.85 (0.71–0.98)	1.30 (1.12–1.48)	1.82 (1.57–2.08)	2.80 (2.34–3.46)	< 0.001
HDL-C (mmol/L)	1.13 ± 0.23	0.96 ± 0.18	0.87 ± 0.15	0.74 ± 0.14	< 0.001
TG/ HDL-C	0.81 (0.63–0.95)	1.38 (1.23–1.52)	2.09 (1.88–2.37)	3.67 (3.09–4.92)	< 0.001
Positive FH-DM	119 (21.06)	95 (17.0)	106 (18.79)	135 (24.15)	0.02
Positive History of CVD	8 (1.42)	15 (2.68)	11 (1.95)	16 (2.86)	0.32
Current smoker	190 (33.63)	224 (40.07)	189 (33.51)	222 (39.71)	0.02
Education					0.42
≤ 6 years	113 (20.0)	101 (18.07)	109 (19.33)	87 (15.56)	
6–12 years	330 (58.41)	333 (59.57)	341 (60.46)	336 (60.11)	
≥ 12 years	122 (21.59)	125 (22.36)	114 (20.21)	136 (24.33)	

Values are mean ± SD or median (IQR) or frequency (%) as appropriate

*N* number, *BMI* body mass index, *WC* waist circumference, *SBP* systolic blood pressure, *DBP* diastolic blood pressure, *FPG* fasting plasma glucose, *2 h-PCG* 2-h post-challenge plasma glucose, *TC* total cholesterol, *TG* triglycerides, *HDL-C* high-density lipoprotein Cholesterol, *FH-DM* family history of diabetes mellitus, *CVD* cardiovascular disease, *NFG* normal fasting glucose, *NGT* normal glucose tolerance, *SD* standard deviation, *IQR* interquartile range

^†^ Normoglycemia: includes NFG and NGT

**Table 2:** Baseline characteristics of the study population according to the TG/HDL-C categories among normoglycemic^†^ women

Variables	First (N = 705)	Second (N = 704)	Third (N = 704)	Forth (N = 704)	*P* Value
	TG/HDL-C < 0.77	0.77 ≤ TG/HDL-C < 1.19	1.19 ≤ TG/HDL-C < 1.84	TG/HDL-C ≥ 1.84	
Age (years)	34.35 ± 12.0	38.27 ± 13.0	40.95 ± 13.1	44.25 ± 12.2	< 0.001
BMI (kg/m^2^)	24.62 ± 4.1	26.66 ± 4.6	27.96 ± 4.4	29.63 ± 4.3	< 0.001
WC (cm)	79.13 ± 10.4	84.45 ± 11.6	88.92 ± 11.4	93.51 ± 10.6	< 0.001
Height (cm)	157.7 ± 5.90	157.10 ± 6.00	156.6 ± 6.00	155.91 ± 5.96	< 0.001
Waist/Height	0.50 ± 0.07	0.54 ± 0.08	0.57 ± 0.08	0.60 ± 0.07	< 0.001
Wrist circumference (cm)	15.36 ± 0.92	15.70 ± 0.94	15.96 ± 0.95	16.30 ± 1.00	< 0.001
SBP (mmHg)	105.2 ± 12.7	107.48 ± 14.4	112.12 ± 16.8	115.11 ± 16.8	< 0.001
DBP (mmHg)	68.90 ± 0.36	70.73 ± 9.11	73.46 ± 9.60	75.90 ± 9.80	< 0.001
FPG (mmol/L)	4.69 ± 0.36	4.78 ± 0.34	4.81 ± 0.36	4.87 ± 0.36	< 0.001
2 h-PCG (mmol/L)	5.16 ± 1.0	5.40 ± 1.00	5.62 ± 1.00	5.96 ± 0.96	< 0.001
TC (mmol/L)	4.36 ± 0.88	4.73 ± 0.97	5.00 ± 1.00	5.27 ± 1.10	< 0.001
TG (mmol/L)	0.71 (0.60–0.85)	1.08 (0.95–1.22)	1.47 (1.28–1.71)	2.26 (1.87–2.77)	< 0.001
HDL-C (mmol/L)	1.33 ± 0.26	1.14 ± 0.20	1.03 ± 0.20	0.86 ± 0.18	< 0.001
TG/ HDL-C	0.57 (0.46–0.68)	0.96 (0.85–1.06)	1.45 (1.31–1.60)	2.54 (2.11–3.33)	< 0.001
Positive FH-DM	100 (14.18)	115 (16.34)	103 (14.63)	97 (13.78)	0.55
Positive History of CVD	5 (0.71)	11 (1.56)	7 (0.99)	14 (1.99)	0.2
Current smoker	36 (5.11)	42 (5.97)	47 (6.68)	44 (6.25)	0.65
Education					< 0.001
≤ 6 years	105 (14.89)	158 (22.44)	205 (29.12)	254(36.08)	
6–12 years	441 (62.55)	421 (59.80)	405 (57.53)	394 (55.97)	
≥ 12 years	159 (22.55)	125 (17.76)	94 (13.35)	56 (7.95)	

Values are mean ± SD or median (IQR) or frequency (%) as appropriate

*N* number, *BMI* body mass index, *WC* waist circumference, *SBP* systolic blood pressure, *DBP* diastolic blood pressure, *FPG* fasting plasma glucose, *2 h-PCG* 2-h post-challenge plasma glucose, *TC* total cholesterol, *TG* triglycerides, *HDL-C* high-density lipoprotein Cholesterol, *FH-DM* family history of diabetes mellitus, *CVD* cardiovascular disease, *NFG* normal fasting glucose, *NGT* normal glucose tolerance, *SD* standard deviation, *IQR* interquartile range

^†^Normoglycemia: includes NFG and NGT

During a median 11.2-year follow-up (IQR: 7.4–12.7 years) of 5064 eligible normoglycemic participants of TLGS (men = 2247 men), 2140 new cases of prediabetes (men = 1070) and 360 incident T2DM (men = 158) were identified. The annual crude incidence rate [95% confidence interval (CI)] of prediabetes in the men population across the first to fourth quartiles of TG/HDL-C was 38.0 (33.3–43.4), 49.4 (43.8–55.7), 51.8 (46.1–58.3), and 56.1 (50.1–62.8) per 1000 person-years, respectively; the corresponding values for women were 23.6 (20.4–27.3), 32.7 (25.5–36.7), 39.4 (35.1–44.3), and 55.8 (50.4–61.8) per 1000 person-years, respectively. Among the normoglycemic population, the annual crude incidence rate (95% CI) of T2DM in the men population across the first to fourth quartiles of TG/HDL-C was 3.7 (2.4–5.5), 5.1 (3.6–7.2), 6.9 (5.2–9.3), and 9.4 (7.3–12.1) per 1000 person-years, respectively; the corresponding values for women were 2.7 (1.8–4.1), 3.7 (2.6–5.3), 7.2 (5.5–9.3), and 11.8 (9.6–14.5) per 1000 person-years, respectively.

From 1414 participants (men = 647) who had prediabetes at baseline, 574 incident cases of T2DM (men = 252) were identified. The annual crude incidence rate (95% CI) of T2DM in the prediabetic men across the first to fourth quartiles of TG/HDL-C was 32.7 (25.1–42.7), 39.1 (30.5–50.2), 39.2 (30.6–50.2), and 47.2 (37.5–59.4) per 1000 person-years, respectively; the corresponding values for women were 30.3 (23.5–39.1), 38.2 (30.3–48.2), 49.6 (40.3–60.8), and 58.0 (47.6–70.6) per 1000 person-years, respectively.

Results of Cox proportional hazard regression analysis exploring the associations between per 1 unit increase in TG/HDL-C ratio and risk of incident prediabetes or diabetes are shown in Table 3. Among women, one unit increase in TG/HDL-C is significantly associated with an increased risk of incident prediabetes or T2DM in normoglycemic and risk of incident T2DM in the prediabetic population. Among normoglycemic men, one unit increase in TG/HDL-C is significantly associated with incident T2DM in all three models. In contrast, for incident prediabetes, only the unadjusted model shows a significant association [HR (95% CI) 1.02 (1.01–1.04)]. Moreover, no associations were found among prediabetic men at baseline in the adjusted models for incident T2DM.

**Table 3:** HRs (95% CIs)^*^ from the multivariable analysis of TG/HDL-C for incident prediabetes^†^, and T2DM^††^

	Men		Women	
	HR (95% CI)	*P* Value	HR (95% CI)	*P* Value
Normoglycemia^†††^ to incident prediabetes (N = 5064)				
Model 1	1.02 (1.01–1.04)	0.001	1.08 (1.07–1.10)	< 0.001
Model 2	1.01 (0.99–1.02)	0.08	1.04 (1.02–1.06)	< 0.001
Model 3	1.01 (0.99–1.02)	0.29	1.04 (1.02–1.06)	< 0.001
Normoglycemia to incident T2DM (N = 5064)				
Model 1	1.07 (1.04–1.11)	< 0.0001	1.12 (1.09–1.15)	< 0.001
Model 2	1.06 (1.02–1.09)	< 0.0001	1.08 (1.04–1.11)	< 0.001
Model 3	1.05 (1.02–1.08)	0.001	1.08 (1.04–1.11)	< 0.001
Prediabetes to incident T2DM (N = 1414)				
Model 1	1.02 (1.00–1.04)	0.04	1.03 (1.01–1.06)	0.004
Model 2	1.01 (0.99–1.03)	0.17	1.02 (1.00–1.04)	0.04
Model 3	1.01 (0.99–1.03)	0.27	1.02 (1.00–1.05)	0.05

Model 1: unadjusted model; Model 2: adjusted for age, body mass index, waist-to-height ratio, wrist circumference, systolic blood pressures, family history of diabetes, education level, history of cardiovascular disease; Model 3: Model 2 + fasting plasma glucose

*HR* hazard ratio, *CI* confidence interval, *TG/HDL-C* triglycerides to high density lipoprotein cholesterol ratio, *T2DM* type 2 diabetes mellitus, *N* number of population, *FPG* fasting plasma glucose, *2 h-PCG* 2-h post-challenge plasma glucose

^*^ HRs (95% CI) for per 1 unit increase in TG/HDL-C

^†^ Predibetes: 5.6 mmol/L ≤ FPG < 7.0 mmol/L or 7.8 mmol/L ≤ 2 h-PCG < 11.1 mmol/L)

^††^ T2DM: FPG ≥ 7.0 mmol/L or 2 h-PCG ≥ 11.1 mmol/L or using antidiabetic medications

^†††^Normoglycemia includes NFG and NGT

Tables 4 and 5 present the HRs (95% CI) of the quartiles of TG/HDL-C (first quartile as reference) for incident prediabetes and T2DM among both normoglycemic and the prediabetic population in three models, for men and women, respectively. Among the normoglycemic population in both sexes, the trend risk of incident prediabetes and T2DM was significant across quartiles in all three models. However, among men, after further adjustment in model 3, the second, third, and fourth quartiles of TG/HDL-C (compared to the first quartile) had HRs (95% CI) of 1.25 (1.05–1.50), 120 (1.00–1.43), and 1.23 (1.03–1.47) with borderline significant association (p for trend = 0.06). Moreover, among prediabetes women, the trend of incident T2DM was significant across quartiles in all three models. By considering prediabetes men, the trend of T2DM risk across quartiles of TG/HDL-C was significant only in model 1 (*P* for trend = 0.03) and reached to nonsignificant level after further adjustment in models 2 and 3.

**Table 4:** HRs (95% CI) of quartiles of TG/HDL-C for incident prediabetes^†^ and T2DM^††^ among men

	Quartiles of TG/HDL				*P* for trend
	First	Second	Third	Fourth	
Normoglycemia^†††^ to incident prediabetes					
	TG/HDL-C < 1.09	1.09 ≤ TG/HDL-C < 1.69	1.69 ≤ TG/HDL-C < 2.65	TG/HDL-C ≥ 2.65	
	(E/N = 219/565)	(E/N = 269/559)	(E/N = 280/564)	(E/N = 302/559)	
Model 1	Reference	1.32 (1.11–1.58)	1.40 (1.17–1.67)	1.50 (1.26–1.78)	< 0.001
Model 2	Reference	1.25 (1.04–1.49)	1.22 (1.02–1.46)	1.29 (1.07–1.54)	0.01
Model 3	Reference	1.25 (1.05–1.50)	1.20 (1.00–1.43)	1.23 (1.03–1.47)	0.06
Normoglycemia to incident T2DM					
	TG/HDL-C < 1.09	1.09 ≤ TG/HDL-C < 1.69	1.69 ≤ TG/HDL-C < 2.65	TG/HDL-C ≥ 2.65	
	(E/N = 23/565)	(E/N = 32/559)	(E/N = 44/564)	(E/N = 59/559)	
Model 1	Reference	1.38 (0.81–2.35)	1.89 (1.14–3.14)	2.53 (1.56–4.10)	< 0.001
Model 2	Reference	1.26 (0.73–2.15)	1.42 (0.85–2.38)	1.84 (1.12–3.01)	0.01
Model 3	Reference	1.26 (0.74–2.17)	1.41 (0.84–2.35)	1.76 (1.07–2.89)	0.02
Prediabetes to incident T2DM					
	TG/HDL-C < 1.35	1.35 ≤ TG/HDL-C < 2.17	2.17 ≤ TG/HDL-C < 3.42	TG/HDL-C ≥ 3.42	
	(E/N = 54/162)	(E/N = 62/162)	(E/N = 63/162)	(E/N = 73/161)	
Model 1	Reference	1.25 (0.87–1.80)	1.25 (0.87–1.79)	1.50 (1.05–2.13)	0.03
Model 2	Reference	1.22 (0.84–1.76)	1.03 (0.70–1.52)	1.32 (0.91–1.91)	0.24
Model 3	Reference	1.29 (0.89–1.87)	1.14 (0.77–1.69)	1.41 (0.97–2.04)	0.12

Model 1: unadjusted model; Model 2: adjusted for age, body mass index, waist-to-height ratio, wrist circumference, systolic blood pressures, family history of diabetes, education level, history of cardiovascular disease; Model 3: Model 2 + fasting plasma glucose

*HR* hazard ratio, *CI* confidence interval, *TG/HDL-C* triglycerides to high density lipoprotein cholesterol ratio, type 2 diabetes mellitus, *E* number of events, *N* number of population, *FPG* fasting plasma glucose, *2 h-PCG* 2-h post-challenge plasma glucose

^† ^Predibetes: 5.6 mmol/L ≤ FPG < 7.0 mmol/L or 7.8 mmol/L ≤ 2 h-PCG < 11.1 mmol/L)

^†† ^T2DM: FPG ≥ 7.0 mmol/L or 2 h-PCG ≥ 11.1 mmol/L or using antidiabetic medications

^†††^Normoglycemia includes NFG and NGT

**Table 5:** HRs (95% CI) of quartiles of TG/HDL-C for incident prediabetes^†^ and T2DM^††^ among women

	Quartiles of TG/HDL-C				*P* for trend
	First	Second	Third	Fourth	
Normoglycemia^†††^ to incident prediabetes					
	TG/HDL-C < 0.77	0.77 ≤ TG/HDL-C < 1.19	1.19 ≤ TG/HDL-C < 1.84	TG/HDL-C ≥ 1.84	
	(E/N = 179/705)	(E/N = 239/704)	(E/N = 283/704)	(E/N = 369/704)	
Model 1	Reference	1.40 (1.15–1.70)	1.69 (1.40–2.04)	2.53 (2.12–3.03)	< 0.001
Model 2	Reference	1.14 (0.94–1.39)	1.20 (0.99–1.46)	1.57 (1.29–1.91)	< 0.001
Model 3	Reference	1.06 (0.87–1.29)	1.13 (0.93–1.37)	1.45 (1.19–1.76)	< 0.001
Normoglycemia to incident T2DM					
	TG/HDL-C < 0.77	0.77 ≤ TG/HDL-C < 1.19	1.19 ≤ TG/HDL-C < 1.84	TG/HDL-C ≥ 1.84	
	(E/N = 22/705)	(E/N = 30/704)	(E/N = 58/704)	(E/N = 92/704)	
Model 1	Reference	1.36 (0.78–2.35)	2.65 (1.62–4.33)	4.52 (2.84–7.19)	< 0.001
Model 2	Reference	1.03 (0.59–1.80)	1.79 (1.08–2.96)	2.55 (1.55–4.18)	< 0.001
Model 3	Reference	0.94 (0.54–1.65)	1.67 (1.00–2.77)	2.36 (1.44–3.87)	< 0.001
Prediabetes to incident T2DM					
	TG/HDL-C < 1.23	1.23 ≤ TG/HDL-C < 1.80	1.80 ≤ TG/HDL-C < 2.81	TG/HDL-C ≥ 2.81	
	(E/N = 60/192)	(E/N = 71/192)	(E/N = 92/192)	(E/N = 99/191)	
Model 1	Reference	1.28 (0.91–1.80)	1.69 (1.22–2.35)	2.00 (1.45–2.76)	< 0.001
Model 2	Reference	1.23 (0.87–1.73)	1.59 (1.14–2.21)	1.86 (1.34–2.58)	< 0.001
Model 3	Reference	1.30 (0.92–1.85)	1.62 (1.16–2.47)	1.84 (1.32–2.55)	< 0.001

Model 1: unadjusted model; Model 2: adjusted for age, body mass index, waist-to-height ratio, wrist circumference, systolic blood pressures, family history of diabetes, education level, history of cardiovascular disease; Model 3: Model 2 + fasting plasma glucose

*HR* hazard ratio, *CI* confidence interval, *TG/HDL-C* triglycerides to high density lipoprotein cholesterol ratio, *T2DM* type 2 diabetes mellitus, *E* number of events, *N* number of population, *FPG* fasting plasma glucose, *2 h-PCG* 2-h post-challenge plasma glucose

^†^ Predibetes: 5.6 mmol/L ≤ FPG < 7.0 mmol/L or 7.8 mmol/L ≤ 2 h-PCG < 11.1 mmol/L)

^††^ T2DM: FPG ≥ 7.0 mmol/L or 2 h-PCG ≥ 11.1 mmol/L or using antidiabetic medications

^†††^ Normoglycemia includes NFG and NGT

As shown in Additional file 1: Table S2 among the whole population, compared to the first quartile, as reference, the second, third, and fourth quartiles were significantly associated with a higher risk of incident prediabetes and T2DM among normoglycemic individuals as well as incident T2DM in the prediabetic population (in all three models *P* for trend < 0.001). The fourth quartile of TG/HDLC was associated with a 37 and 92% higher risk of incident prediabetes and T2DM among the normoglycemic population and a 57% higher risk for incident T2DM in the prediabetic population.

In the sensitivity analysis among women with insulin data (NFG/NGT = 1705 and prediabetes = 422), similar results to the original population were observed when we included HOMA-IR in place of FPG in our data analysis (Additional file 1: Table S3). Although, among men, no significant association was observed either for incident prediabetes or T2DM in the normoglycemic (n = 1192) or the prediabetic population (n = 339).

## Discussion

This study examined the association between TG/HDL-C and incident prediabetes and T2DM after adjustment for age, BMI, waist-to-height ratio, wrist circumference, SBP, FH-DM, education level, history of CVD, and FPG level in a prospective cohort study with a long duration of follow-up in the Middle East and North Africa (MENA) region. Generally, we found among the whole population with NFG/NGT, an increasing value of TG/HDL-C, whether as a continuous or categorical variable, was significantly associated with incident prediabetes and T2DM; this issue was more prominent among women. In our data analysis, a similar association between TG/HDL-C and incident T2DM was also demonstrated in individuals with prediabetes; however, this relationship was found only among women in sex-stratified analysis. Generally, the associations between TG/HDL-C and incident prediabetes and T2DM persisted even after adjustment for HOMA-IR in place of FPG among women.

Similar to our study, a growing body of cohort studies demonstrated an independent association between TG/HDL-C and incident T2DM. Notably, different sources of heterogeneity limit the comparison of our findings with other studies in this field, including (1) study design (retrospective vs. prospective cohorts); (2) level of adjustment for confounders; (3) sex-stratified vs. whole population analysis; (4) age of the study population, and (5) follow-up duration. Furthermore, about 77.8% (14 out of 18 cohort studies) were reported from the East Asian populations, and only a few were conducted in other parts of the world.

Mackey et al. in a Multi-Ethnic Study of Atherosclerosis (MESA) with a mean 7.7 years follow-up, showed that a higher TG/HDL-C was associated with incident T2DM after adjustment for well-known confounders. Considering IR indices, this association persisted after adjustment for HOMA-IR, while adjusting for another IR index named lipoprotein-based IR index (LP-IR), an index that combines lipoprotein parameters, weighted by the strength of correlations with HOMA-IR made this association to be disappeared. [31]. In the current study, we also demonstrated that the strong association between TG/HDL-C and incident prediabetes and T2DM (excluding those converting from prediabetes to T2DM) did not change with adjustment for HOMA-IR in the whole population, the relationship that mainly attributed to the female sex. Janghorbani et al., in a prospective cohort study conducted in a high-risk Iranian population with positive FH-DM in Isfahan province, did not find any association between TG/HDL-C and incident T2DM during seven years of follow-up, even in the age and sex-adjusted analysis [32]. Since there is a strong association between FH-DM and incident T2DM, their results cannot be generalized to the general population. Furthermore, compared to our study, Janghorbani et al. study had a shorter duration of follow-up. Another recent study conducted in a large-scale prospective community-based Korean cohort study showed that among middle-aged individuals in more than a decade follow-up, similar to our study, TG/HDL-C in a dose-dependent manner had a significant association with incident T2DM in the multivariable model adjusted for main confounders including HOMA-IR [33]. A multiethnic cohort from the Insulin Resistance Atherosclerosis (IRAS) Family Study with about five years of follow-up showed that similar to Caucasians, TG/HDL-C could be used to identify IR in Hispanics and African Americans. This study showed that TG/HDL-C might efficiently predict T2DM just in Hispanic and African-American women; however, by adjustment for glucose disposition index, i.e., an index that includes insulin sensitivity and amount of insulin secreted in response to blood glucose levels, this association reached null. The authors indicated the role of impaired β-cell function on the link between TG/HDL-C and incident T2DM [26]. We previously reported a strong association between TG/HDL-C and incident IR [34] and diabetes both in medium (6 years) and long-term follow-up in a non-diabetic Iranian population [17, 21]. In the current study, we extended our previous research by showing the independent role of TG/HDL-C in the development of both prediabetes and T2DM among normoglycemic Tehranian adults, even in the presence of HOMA-IR. Moreover, in line with a recent cohort and meta-analysis conducted by Cheng et al. [10], we found that the impact of TG/HDL-C on incident prediabetes and T2DM was generally more substantial among women than men.

To our knowledge, the present study is the first to show the impact of TG/HDL-C on the development of T2DM among prediabetic individuals, the association that was mainly found in women. Moreover, this relationship tends to be reached a significant level even in the presence of HOMA-IR in women. We found only one retrospective cohort study conducted in China that investigated the association between TG/HDL-C and incident T2DM in prediabetic subjects. The authors reported that individuals with prediabetes in the highest TG/HDL-C quartile had 40% higher risk than the reference group in the multivariate analysis [35]; the corresponding value in our study was about 80%.

Besides the prospective cohort studies, the association between TG/HDL-C with incident T2DM was also examined in the retrospective cohorts. Gong et al., in a retrospective cohort study of Chinese individuals, reported a positive correlation between TG/HDL-C and incident prediabetes and T2DM. The researchers, however, defined prediabetes (i.e., IGT) as patients with diabetes whose FPG ≥ 6 mmol/L or self-reported rather than based on the oral glucose tolerance test [20]. Another large retrospective cohort study among the Korean population showed a dose–response significant association between TG/HDL-C and risk of new-onset diabetes in both sexes. [36].

Potential mechanisms may contribute to the association between TG/HDL-C and incident prediabetes and T2DM. Recently, a positive correlation between TG levels and IR in a normal glucose tolerance state and a negative correlation with beta-cell function in people with dyslipidemia were reported. These associations may be mediated through the independent influence of TG on insulin secretion and the decrement of superoxide dismutase, a primary antioxidant enzyme in the body, followed by a higher level of oxidative stress and beta-cell damage [37]. Higher TG level results in increased free fatty acids that contribute to higher TG and a decrease in HDL-C, an issue that leads to a higher TG/HDL-C ratio [38]. HDL-C has diverse functions in pancreatic beta-cell regulation, such as its effect on cellular lipid accumulation, endoplasmic reticulum stress, and apoptosis. Pancreatic lipid accumulation and following toxicity lead to the inhibition of insulin secretion. Moreover, HDL-C may affect insulin secretion through action on insulin transcription [39].

As strengths, this study was conducted in the context of the most significant ongoing cohort of the MENA region with a reasonably long follow-up. Furthermore, in the current study, we applied well-controlled measurements rather than a self-reporting approach for collecting data on anthropometric indices, blood pressure, and different states of dysglycemia. Additionally, all laboratory measurements were performed using standardized methods and monitored by strict quality control protocols. As another strength, we included HOMA-IR in our data analysis. The current study has some potential limitations. First, we measured HDL-C and TG only once at baseline, an issue that could lead to regression dilution bias. Second, a single baseline lipid measurement did not consider the significant intra-individual variation in TG level and changes in serum lipid levels during the follow-up time. Third, we did not measure hemoglobin A1C as another criterion for defining prediabetes and T2DM. Fourth, we did not consider nutritional data in our analysis. Finally, this study was conducted in the metropolitan city of Tehran; hence, its results cannot be extrapolated to the rural regions.

In conclusion, in normoglycemic individuals, increasing the value of TG/HDL-C was unfavorably associated with incident prediabetes and T2DM, especially among women. Similarly, this lipid ratio was also associated with incident T2DM in the prediabetic population. Generally, we found that the correlation between TG/HDL-C and different states of dysglycemia is independent of IR states. Hence, lowering TG/HDL-C might potentially prevent prediabetes and T2DM, but the issue needs further investigation.

## Supplementary Information

**Additional file 1. Table S1** Baseline characteristics of the study population by respondents and non-respondents: Tehran Lipid and Glucose Study.

**Additional file 2. Table S2** HRs (95% CIs)^*^ of TG/HDL-C for incident prediabetes^†^ and T2DM^††^ among total population.

**Additional file 3. Table S3** HRs (95% CIs) of TG/HDL-C for incident prediabetes and T2DM among a population with HOMA-IR data.

## Data Availability

The datasets used and/or analyzed in the study are available from the corresponding author upon reasonable request.

## Abbreviations

DM: Diabetes mellitus
T2DM: Type 2 diabetes mellitus
IFG: Impaired fasting glucose
IGT: Impaired glucose tolerance
HbA1C: Glycated hemoglobin
CVD: Cardiovascular diseases
IR: Insulin resistance
HOMA-IR: Homeostasis model assessment of insulin resistance
TG: Triglycerides
HDL-C: High-density lipoprotein cholesterol
TLGS: Tehran lipid and glucose study
NFG: Normal fasting glucose
NGT: Normal glucose tolerance
FPG: Fasting plasma glucose
2 h-PCG: 2-Hour post-challenge plasma glucose
RIES: Research Institute for Endocrine Sciences
FH-DM: Family history of diabetes mellitus
BMI: Body mass index
WC: Waist circumference
SBP: Systolic blood pressure
DBP: Diastolic blood pressure
TC: Total cholesterol
CV: Coefficient of variation
SD: Standard deviation
IQR: Interquartile range
HR: Hazard ratio
CI: Confidence interval
MENA: Middle East and North Africa
MESA: Multi-Ethnic Study of Atherosclerosis
LP-IR: Lipoprotein-based insulin resistance index
IRAS: Insulin resistance atherosclerosis

## References

[1] Duan D, Kengne AP, Echouffo-Tcheugui JB (2021). Screening for diabetes and prediabetes. Endocrinol Metab Clin.

[2] Ogurtsova K, da Rocha FJ, Huang Y, Linnenkamp U, Guariguata L, Cho NH, Cavan D, Shaw J, Makaroff L (2017). IDF diabetes atlas: global estimates for the prevalence of diabetes for 2015 and 2040. Diabetes Res Clin Pract.

[3] Derakhshan A, Sardarinia M, Khalili D, Momenan AA, Azizi F, Hadaegh F (2014). Sex specific incidence rates of type 2 diabetes and its risk factors over 9 years of follow-up: Tehran Lipid and Glucose Study. PLoS ONE.

[4] Draznin B, Aroda VR, Bakris G, Benson G, Brown FM, Freeman R, Green J, Huang E, Isaacs D, Kahan S (2022). 2. Classification and diagnosis of diabetes standards of medical care in diabetes-2022. Diabetes.

[5] Saeedi P, Petersohn I, Salpea P, Malanda B, Karuranga S, Unwin N, Colagiuri S, Guariguata L, Motala AA, Ogurtsova K (2019). Global and regional diabetes prevalence estimates for 2019 and projections for 2030 and 2045: results from the International Diabetes Federation Diabetes Atlas. Diabetes Res Clin Pract.

[6] Esteghamati A, Gouya MM, Abbasi M, Delavari A, Alikhani S, Alaedini F, Safaie A, Forouzanfar M, Gregg EW (2008). Prevalence of diabetes and impaired fasting glucose in the adult population of Iran: national survey of risk factors for non-communicable diseases of Iran. Diabetes Care.

[7] Hadaegh F, Derakhshan A, Zafari N, Khalili D, Mirbolouk M, Saadat N, Azizi F (2017). Pre-diabetes tsunami: incidence rates and risk factors of pre-diabetes and its different phenotypes over 9 years of follow-up. Diabet Med.

[8] Whaley-Connell A, Pavey BS, McCullough PA, Saab G, Li S, McFarlane SI, Chen SC, Vassalotti JA, Collins AJ, Bakris G (2010). Dysglycemia predicts cardiovascular and kidney disease in the Kidney early evaluation program. J Clin Hypertens.

[9] Ares J, Valdés S, Botas P, Sánchez-Ragnarsson C, Rodríguez-Rodero S, Morales-Sanchez P, Menéndez-Torre E, Delgado E (2019). Mortality risk in adults according to categories of impaired glucose metabolism after 18 years of follow-up in the North of Spain: the asturias study. PLoS ONE.

[10] Cheng C, Liu Y, Sun X, Yin Z, Li H, Zhang M, Zhang D, Wang B, Ren Y, Zhao Y (2019). Dose–response association between the triglycerides: High-density lipoprotein cholesterol ratio and type 2 diabetes mellitus risk: the rural Chinese cohort study and meta-analysis. J Diabetes.

[11] Ghasemi A, Tohidi M, Derakhshan A, Hasheminia M, Azizi F, Hadaegh F (2015). Cut-off points of homeostasis model assessment of insulin resistance, beta-cell function, and fasting serum insulin to identify future type 2 diabetes: Tehran lipid and glucose study. Acta Diabetol.

[12] Tohidi M, Baghbani-Oskouei A, Ahanchi NS, Azizi F, Hadaegh F (2018). Fasting plasma glucose is a stronger predictor of diabetes than triglyceride–glucose index, triglycerides/high-density lipoprotein cholesterol, and homeostasis model assessment of insulin resistance: Tehran Lipid and Glucose Study. Acta Diabetol.

[13] Zhou M, Zhu L, Cui X, Feng L, Zhao X, He S, Ping F, Li W, Li Y (2016). The triglyceride to high-density lipoprotein cholesterol (TG/HDL-C) ratio as a predictor of insulin resistance but not of β cell function in a Chinese population with different glucose tolerance status. Lipids Health Dis.

[14] Chiang JK, Lai NS, Chang JK, Koo M (2011). Predicting insulin resistance using the triglyceride-to-high-density lipoprotein cholesterol ratio in Taiwanese adults. Cardiovasc Diabetol.

[15] Salazar MR, Carbajal HA, Espeche WG, Leiva Sisnieguez CE, Balbín E, Dulbecco CA, Aizpurúa M, Marillet AG, Reaven GM (2012). Relation among the plasma triglyceride/high-density lipoprotein cholesterol concentration ratio, insulin resistance, and associated cardio-metabolic risk factors in men and women. Am J Cardiol.

[16] Guo W, Qin P, Lu J, Li X, Zhu W, Xu N, Wang J, Zhang Q (2019). Diagnostic values and appropriate cutoff points of lipid ratios in patients with abnormal glucose tolerance status: a cross-sectional study. Lipids Health Dis.

[17] Bozorgmanesh M, Hadaegh F, Ghaffari S, Harati H, Azizi F (2011). A simple risk score effectively predicted type 2 diabetes in Iranian adult population: population-based cohort study. The European Journal of Public Health.

[18] von Bibra H, Saha S, Hapfelmeier A, Müller G, Schwarz PE (2017). Impact of the triglyceride/high-density lipoprotein cholesterol ratio and the hypertriglyceremic-waist phenotype to predict the metabolic syndrome and insulin resistance. Horm Metab Res.

[19] Çin NNA, Yardımcı H, Koç N, Uçaktürk SA, Ok MA (2020). Triglycerides/high-density lipoprotein cholesterol is a predictor similar to the triglyceride–glucose index for the diagnosis of metabolic syndrome using International Diabetes Federation criteria of insulin resistance in obese adolescents: a cross-sectional study. J Pediatr Endocrinol Metab.

[20] Gong R, Liu Y, Luo G, Liu W, Jin Z, Xu Z, Li Z, Yang L, Wei X (2021). Associations of TG/HDL ratio with the risk of prediabetes and diabetes in Chinese adults a Chinese population cohort study based on open data. In tJ Endocrinol.

[21] Hadaegh F, Hatami M, Tohidi M, Sarbakhsh P, Saadat N, Azizi F (2010). Lipid ratios and appropriate cut off values for prediction of diabetes: a cohort of Iranian men and women. Lipids Health Dis.

[22] Nguyen HH, Tran HH, Nguyen LT, Nguyen T, Nguyen NA, Vi MT, Nguyen KT (2022). TG/HDL-C ratio is a risk factor associated with CKD: use in assessing the risk of progression of CKD. Pathophysiology.

[23] Sato F, Nakamura Y, Kayaba K, Ishikawa S (2022). TG/HDL-C ratio as a predictor of stroke in the population with healthy BMI: the Jichi medical school cohort study. Nut Metab Cardiovasc Dis.

[24] Kiyosue A (2018). Nonfasting TG/HDL-C ratio seems a good predictor of MACE in CAD patients with statin therapy Could it be a treatment target?. J Cardiol.

[25] Jialal I, Adams-Huet B, Remaley AT (2022). A comparison of the ratios of C-reactive protein and triglycerides to high-density lipoprotein-cholesterol as biomarkers of metabolic syndrome in African Americans and non-Hispanic Whites. J Diabetes Complications.

[26] Young KA, Maturu A, Lorenzo C, Langefeld CD, Wagenknecht LE (2019). Chen Y-DI, Taylor KD, Rotter JI, Norris JM, Rasouli N: the triglyceride to high-density lipoprotein cholesterol (TG/HDL-C) ratio as a predictor of insulin resistance, β-cell function, and diabetes in Hispanics and African Americans. J Diabetes Complications.

[27] Chen Y, Liu Y, Zhao Y, Fu J, Zhang Y, Liu Y, Fan Z (2021). Triglyceride to high-density lipoprotein cholesterol ratio and cardiovascular events in the general population: a systematic review and meta-analysis of cohort studies. Nutr Metab Cardiovasc Dis.

[28] Azizi F, Madjid M, Rahmani M, Emami H, MIRMIRAN P, Hadjipour R. 2000 Tehran Lipid and Glucose Study (TLGS): rationale and design.

[29] Muniyappa R, Lee S, Chen H, Quon MJ (2008). Current approaches for assessing insulin sensitivity and resistance in vivo: advantages, limitations, and appropriate usage. Am J Physiol Endocrinol Metab.

[30] Jahangiri Noudeh Y, Hadaegh F, Vatankhah N, Momenan AA, Saadat N, Khalili D, Azizi F (2013). Wrist circumference as a novel predictor of diabetes and prediabetes: results of cross-sectional and 88-year follow-up studies. J Clin Endocrinol Metab.

[31] Mackey RH, Mora S, Bertoni AG, Wassel CL, Carnethon MR, Sibley CT, Goff DC (2015). Lipoprotein particles and incident type 2 diabetes in the multi-ethnic study of atherosclerosis. Diabetes Care.

[32] Janghorbani M, Amini M (2016). Utility of serum lipid ratios for predicting incident type 2 diabetes: the Isfahan diabetes prevention study. Diabetes Metab Res Rev.

[33] Lim T-K, Lee HS, Lee Y-J (2020). Triglyceride to HDL-cholesterol ratio and the incidence risk of type 2 diabetes in community dwelling adults: a longitudinal 12-year analysis of the Korean Genome and Epidemiology Study. Diabetes Res Clin Pract.

[34] Derakhshan A, Tohidi M, Hajebrahimi M, Saadat N, Azizi F, Hadaegh F (2017). Sex-specific incidence rates and risk factors of insulin resistance and β–cell dysfunction: a decade follow-up in a Middle Eastern population. Diabet Med.

[35] Sun Y, Wang Z, Huang Z, Hu H, Han Y (2022). The association between the triglyceride-to-high-density lipoprotein cholesterol ratio and the risk of progression to diabetes From prediabetes: a 5-year cohort study in chinese adults. Front endocrinol.

[36] Kim J, Shin S-J, Kim Y-S, Kang H-T (2021). Positive association between the ratio of triglycerides to high-density lipoprotein cholesterol and diabetes incidence in Korean adults. Cardiovasc Diabetol.

[37] Ma M, Liu H, Yu J, He S, Li P, Ma C, Zhang H, Xu L, Ping F, Li W (2020). Triglyceride is independently correlated with insulin resistance and islet beta cell function: a study in population with different glucose and lipid metabolism states. Lipids Health Dis.

[38] Jung UJ, Choi M-S (2014). Obesity and its metabolic complications: the role of adipokines and the relationship between obesity, inflammation, insulin resistance, dyslipidemia and nonalcoholic fatty liver disease. Int J Mol Sci.

[39] Siebel AL, Heywood SE, Kingwell BA (2015). HDL and glucose metabolism: current evidence and therapeutic potential. Front Pharmacol.

