# Supplementary material for: Association of triglycerides to high-density lipoprotein cholesterol ratio to identify future prediabetes and type 2 diabetes mellitus: over one-decade follow-up in the Iranian population
Source: Diabetol Metab Syndr. 2023 Feb 2;15:13. doi: 10.1186/s13098-023-00988-0 (PMC9893691; doi:10.1186/s13098-023-00988-0)
Supplement: Supplementary file 1 — Additional file 1. Table S1 Baseline characteristics of the study population by respondents and non-respondents: Tehran Lipid and Glucose Study. [file 13098_2023_988_MOESM1_ESM.docx]

| **Supplementary Table 1** Baseline characteristics of the study population by respondents and non-respondents: Tehran Lipid and Glucose Study | | | |
| --- | --- | --- | --- |
| **Variables** | **Respondents**  **(N = 7877)** | **Non-respondents**  **(N = 1392)** | ***P* Value** |
| Age (years) | 40.5 ± 13.9 | 37.0 ± 16.2 | < 0.0001 |
| BMI (kg/m^2^) | 26.6 ± 4.5 | 25.8 ± 5.4 | < 0.0001 |
| WC (cm) | 88.7 ± 11.8 | 86.3 ± 13.5 | < 0.0001 |
| Height (cm) | 163.2 ± 9.6 | 163.4 ± 9.8 | 0.5915 |
| Waist/Height | 0.54 ± 0.08 | 0.53 ± 0.09 | < 0.0001 |
| Wrist circumference (cm) | 16.7 ± 1.4 | 16.5 ± 1.6 | 0.0002 |
| SBP (mmHg) | 112.8 ± 15.9 | 112.0 ± 18.2 | 0.1431 |
| DBP (mmHg) | 73.3 ± 10.1 | 73.1 ± 10.6 | 0.6799 |
| FPG (mmol/L) | 4.83± 0.36 | 4.79 ± 0.37 | 0.0009 |
| 2h-PCG (mmol/L) | 5.43 ± 1.11 | 5.35 ± 1.11 | 0.1000 |
| TC (mmol/L) | 4.81 ± 1.02 | 4.59 ± 1.00 | < 0.0001 |
| TG (mmol/L) | 1.37 (0.96-1.97) | 1.21 (0.87-1.80) | < 0.0001 |
| HDL-C (mmol/L) | 1.02 ± 0.29 | 1.05 ± 0.27 | 0.0003 |
| TG/ HDL-C | 1.37 (0.87-2.20) | 1.22 (0.78-1.92) | < 0.0001 |
| Positive FHDM | 870 (17.2) | 210 (15.1) | 0.065 |
| Positive History of CVD | 87 (1.7) | 26 (1.9) | 0.703 |
| Current smoker | 994 (19.6) | 271 (23.0) | 0.011 |
| Education |  |  | 0.233 |
| ≤ 6 years | 1132 (22.4) | 300 (21.7) |  |
| 6 - 12 years | 3001 (59.3) | 852 (61.6) |  |
| ≥ 12 years | 931 (18.4) | 231 (16.7) |  |
| Values are mean ± SD or median (IQR) or frequency (%) as appropriate.  *BMI*  body mass index, *WC* waist circumference, *SBP* systolic blood pressure, *DBP* diastolic blood pressure, *FPG* fasting plasma glucose, *2h-PCG* 2-hour post-challenge plasma glucose, *TC* total cholesterol, *TG* triglycerides, *HDL-C* high-density lipoprotein Cholesterol, *FH-DM* family history of diabetes, *CVD* cardiovascular disease, *SD* standard deviation, *IQR* interquartile range | | | |
