# Supplementary material for: Association of triglycerides to high-density lipoprotein cholesterol ratio to identify future prediabetes and type 2 diabetes mellitus: over one-decade follow-up in the Iranian population
Source: Diabetol Metab Syndr. 2023 Feb 2;15:13. doi: 10.1186/s13098-023-00988-0 (PMC9893691; doi:10.1186/s13098-023-00988-0)
Supplement: Supplementary file 2 — Additional file 2. Table S2 HRs (95% CIs)* of TG/HDL-C for incident prediabetes† and T2DM†† among total population. [file 13098_2023_988_MOESM2_ESM.docx]

| Supplementary Table 2. HRs (95% CIs)^*^ of TG/HDL-C for incident prediabetes^†^ and T2DM^††^ among total population | | | | | | | | | |
| --- | --- | --- | --- | --- | --- | --- | --- | --- | --- |
|  | **Quartiles of TG/HDL-C** | | | | | | | | **Per 1 unit increase in TG/HDL-C** |
| Normoglycemia^†††^ to incident prediabetes (N = 5064) | | | | | | | | | |
|  | **First**  TG/HDL-C < 0.87  (E/N = 372 / 1,267) |  | **Second**  0.87 ≤ TG/HDL-C < 1.37  (E/N = 496 / 1,265) |  | **Third**  1.37 ≤ TG/HDL-C < 2.20  (E/N = 601 / 1,266) |  | **Fourth**  TG/HDL-C ≥ 2.20  (E/N = 671 / 1,266) | ***P***  **for trend** | **HR (95% CI)** |
| Model 1 | Reference |  | 1.41 (1.23-1.62) |  | 1.87 (1.64-2.13) |  | 2.12 (1.87-2.41) | < 0.001 | 1.05 (1.04-1.06) |
| Model 2 | Reference |  | 1.17 (1.02-1.34) |  | 1.39 (1.21-1.59) |  | 1.44 (1.22-1.65) | < 0.001 | 1.02 (1.01-1.03) |
| Model 3 | Reference |  | 1.14 (1.00-1.31) |  | 1.33 (1.16-1.53) |  | 1.37 (1.20-1.58) | < 0.001 | 1.02 (1.00-1.03) |
|  | | | | | | | | | |
| Normoglycemia to incident T2DM (N = 5064) | | | | | | | | | |
|  | TG/HDL-C < 0.87  (E/N =44 / 1,267) |  | 0.87 ≤ TG/HDL-C < 1.37  (E/N = 66 / 1,265) |  | 1.37 ≤ TG/HDL-C < 2.20  (E/N = 113 / 1,266) |  | TG/HDL-C ≥ 2.20  (E/N = 137 / 1,266) | ***P***  **for trend** | **HR (95% CI)** |
| Model 1 | Reference |  | 1.48 (1.01-2.17) |  | 2.64 (1.86-3.74) |  | 3.19 (2.27-4.48) | < 0.001 | 1.09 (1.07-1.11) |
| Model 2 | Reference |  | 1.18 (0.80-1.74) |  | 1.83 (1.28-2.62) |  | 1.99 (1.39-2.86) | < 0.001 | 1.06 (1.04-1.09) |
| Model 3 | Reference |  | 1.14 (0.77-1.67) |  | 1.77 (1.23-2.53) |  | 1.92 (1.34-2.75) | < 0.001 | 1.06 (1.03-1.08) |
|  | | | | | | | | | |
| Prediabetes to incident T2DM (N = 1414) | | | | | | | | | |
|  | TG/HDL-C < 1.28  (E/N = 112 / 354) |  | 1.28 ≤ TG/HDL-C < 1.96  (E/N = 145 / 353) |  | 1.96 ≤ TG/HDL-C < 3.10  (E/N = 149 / 354) |  | TG/HDL-C ≥ 3.10  (E/N = 168 / 353) | ***P***  **for trend** | **HR (95% CI)** |
| Model 1 | Reference |  | 1.42 (1.11-1.82) |  | 1.46 (1.14-1.86) |  | 1.70 (1.34-2.16) | < 0.001 | 1.02 (1.01-1.04) |
| Model 2 | Reference |  | 1.36 (1.06-1.74) |  | 1.37 (1.07-1.76) |  | 1.54 (1.20-1.97) | < 0.001 | 1.02 (1.00-1.03) |
| Model 3 | Reference |  | 1.42 (1.11-1.82) |  | 1.47 (1.14-1.88) |  | 1.57 (1.22-2.01) | < 0.001 | 1.01 (1.00-1.03) |
| * HRs for per 1 unit increase in TG/HDL-C  † Predibetes: 5.6 mmol/L≤ FPG < 7.0 mmol/L or 7.8 mmol/L ≤ 2h-PCG < 11.1 mmol/L)  †† T2DM: FPG ≥ 7.0 mmol/L or 2h-PCG ≥ 11.1 mmol/L or using antidiabetic medications  ††† Normoglycemia includes NFG and NGT.  Model 1: unadjusted model, Model 2: adjusted for sex, age, body mass index, waist-to-height ratio, wrist circumference, systolic blood pressures, family history of diabetes, education levels, history of cardiovascular disease, Model 3: Model 2 + fasting plasma glucose  *HR* hazard ratio, *CI* confidence interval, *TG/HDL-C* triglycerides to high density lipoprotein cholesterol ratio, *T2DM* type 2 diabetes mellitus, *E* number of events, *N* number of population, *FPG* fasting plasma glucose, *2h-PCG* 2-hour post-challenge plasma glucose | | | | | | | | | |
