# Supplementary material for: Association of triglycerides to high-density lipoprotein cholesterol ratio to identify future prediabetes and type 2 diabetes mellitus: over one-decade follow-up in the Iranian population
Source: Diabetol Metab Syndr. 2023 Feb 2;15:13. doi: 10.1186/s13098-023-00988-0 (PMC9893691; doi:10.1186/s13098-023-00988-0)
Supplement: Supplementary file 3 — Additional file 3. Table S3 HRs (95% CIs) of TG/HDL-C for incident prediabetes and T2DM among a population with HOMA-IR data. [file 13098_2023_988_MOESM3_ESM.docx]

| **Supplementary Table 3**: HRs (95% CIs) of TG/HDL-C for incident prediabetes and T2DM among a population with HOMA-IR data | | | | | | | | | |
| --- | --- | --- | --- | --- | --- | --- | --- | --- | --- |
|  | **Men** | |  | **Women** | |  | **Total population** | | |
|  | **HR (95% CI)** | ***P* Value** |  | **HR (95% CI)** | ***P* Value** |  | **HR (95% CI)** | | ***P* Value** |
| **Normoglycemia^†††^ to incident prediabetes** | (E/N = 623 / 1,192) |  |  | (E/N = 723 / 1,705) |  |  | (E/N = 1,346 / 2,897) | |  |
| Model 1 | 1.02 (1.00-1.04) | 0.01 |  | 1.09 (1.06-1.11) | < 0.001 |  | 1.05 (1.04-1.07) | | < 0.001 |
| Model 2 | 1.02 (0.99-1.04) | 0.06 |  | 1.04 (1.02-1.07) | <0.001 |  | 1.02 (1.01-1.04) | | 0.001 |
|  | | | | | | | | | |
| **Normoglycemia to incident T2DM** | (E/N = 97 / 1,192) |  |  | (E/N = 130 / 1,705) |  |  | (E/N = 227 / 2,897) |  | |
| Model 1 | 1.04 (0.99-1.08) | 0.11 |  | 1.13 (1.08-1.17) | < 0.001 |  | 1.07 (1.04-1.10) | < 0.001 | |
| Model 2 | 1.02 (0.97-1.07) | 0.39 |  | 1.08 (1.03-1.14) | 0.001 |  | 1.03 (1.00-1.07) | 0.06 | |
|  | | | | | | | | | |
| **Prediabetes to incident T2DM** | (E/N = 129 / 339) |  |  | (E/N = 183 / 422) |  |  | (E/N = 312 / 761) |  | |
| Model 1 | 1.02 (0.98-1.05) | 0.38 |  | 1.04 (1.00-1.07) | 0.03 |  | 1.02 (1.00-1.05) | 0.05 | |
| Model 2 | 1.00 (0.96-1.05) | 0.81 |  | 1.03 (0.99-1.06) | 0.06 |  | 1.01 (0.99-1.04) | 0.29 | |
| † Predibetes: 5.6 mmol/L≤ FPG < 7.0 mmol/L or 7.8 mmol/L ≤ 2h-PCG < 11.1 mmol/L)  †† T2DM: FPG ≥ 7.0 mmol/L or 2h-PCG ≥ 11.1 mmol/L or using antidiabetic medications  ††† Normoglycemia includes NFG and NGT.  * HRs for per 1 unit increase in TG/HDL-C  Model 1: unadjusted model, Model 2: adjusted for age, body mass index, waist-to-height ratio, wrist circumference, systolic blood pressures, family history of diabetes, education levels, history of cardiovascular disease, HOMA-IR  *HR* hazard ratio, CI, confidence interval, *TG/HDL-C* triglycerides to high-density lipoprotein cholesterol ratio, *T2DM* type 2 diabetes mellitus, *HOMA-IR* homeostasis model assessment of insulin resistance*, E* number of events, *N* number of population | | | | | | | | | |
